# Spaces and Places for Connection in the Postdigital University

**DOI:** 10.1007/s42438-022-00317-0

**Published:** 2022-06-18

**Authors:** Karen Gravett, Patrick Baughan, Namrata Rao, Ian Kinchin

**Affiliations:** 1 Surrey Institute of Education, University of Surrey, Guildford, Surrey GU2 7XH UK; 2 Advance HE, York, UK; 3 School of Education, Liverpool Hope University, Liverpool, UK

**Keywords:** Spaces and places, Postdigital university, Teacher learning, Post-pandemic, Sociomaterial, Photovoice method

## Abstract

This study focuses on the spaces and places for learning and teaching connections in higher education. Using a photovoice research method, we ask: what role do spaces and places play in offering opportunities for learning and teaching connection, and what do they tell us about the evolving practices of teachers in contemporary higher education? Whilst considerable attention has been paid to the learning spaces of students, we argue that less attention has been devoted to the spaces in which educators learn. Our findings are considered against a backdrop of the ongoing disruption of the Covid-19 pandemic, meaning that opportunities for interaction have assumed even greater significance, and the ways in which we use and understand teaching spaces are in flux. As such, our data highlights how the move to digital and hybrid learning is blurring the boundaries of spaces and places, reorienting what it means to teach and to learn in a postdigital higher education landscape. We engage sociomaterial and spatial concepts to examine how spaces entangle with university teachers’ experiences, and we explore the shifting nature of interaction and space in post-pandemic times.

## Introduction

Where and in what spaces do teachers learn and encounter connection? In this article, we employ an approach inspired by the creative and participatory photovoice method, to examine the occluded opportunities for learning and teaching encounters, and the spaces in which such encounters might occur. We consider what role interactions, with students and colleagues, might play in teachers’ lives, in what physical spaces such connections unfold, and how such encounters might sustain us in troubled times. Our research had three aims. Firstly, we sought to explore the spaces and places that enable connections in learning and teaching. At the same time, we were interested in examining how such moments support teachers’ development and well-being. Finally, we were interested to understand the complexity and sociomateriality of educational practices, and specifically how such spaces and practices might be evolving in view of the altered higher education landscape.

Our study took place during the ongoing disruption following the imposition of national lockdowns in many parts of the world, and the rapid ‘pivot’ to remote learning and teaching within university education. This had several important implications. Opportunities for interaction assumed greater significance and relational interactions offered escapes and opportunities for support. And yet, such connections were complicated by our inability to connect face to face, as well as reorientated by the myriad of ways that are emerging for connections within a postdigital world. As such, our article examines the implications of the reshaping of working practices for contemporary teachers. We employ the notion of postdigital to indicate a time when digital learning has become ubiquitous, where technologies are integrated and fundamental to our everyday lives, and where binary juxtapositions between ‘in person’ and online are no longer meaningful (Gourlay 2021; Fawns 2019; Jandrić et al. 2018). Through participants’ reflections and photographs, we glean insight into the larger stories of working as a teacher in the contemporary university.

## Conversations and Connections in Higher Education

Education literature has long foregrounded the value of informal connections for learning (Brookfield 1995; Roxå and Mårtensson 2009; Thomson and Trigwell 2018; Thomson and Barrie 2021; Cook-Sather, Hong, Moss, and Williamson 2021), as well as the role of reflective practice (Brookfield 1995; Ashwin 2015). Research has surfaced the ways in which dialogic connections serve to develop teachers and their practices (McCormack and Kennelly 2011; Cook-Sather, Hong, Moss, and Williamson 2021). Roxå and Mårtensson (2009) explore teachers’ networks, where informal conversations provide a basis for meaningful learning. For Brookfield (1995), one of the key pillars of the reflective process is dialogue and connection with others. Similarly, research undertaken by Bell and Thompson (2018) and Czerniawski et al. (2017) highlight the importance of colleagues’ informal interactions. Hartung and Wilson (2016) explore how cross-organisational ‘learning conversations’ offer an important source of learning (see also Eraut 2004). Such learning opportunities can foster a sense of community where individuals feel supported (Czerniawski et al. 2017). Evidently, meaningful interactions may hold valuable potential to influence both teaching practice and teachers’ well-being, offering openings within the ‘cracks’ of marketised higher education (Bottrell and Manathunga 2019). And yet, whilst much has been written about reflective conversations, less attention has been devoted to understanding the spatiality and materiality of such encounters.

## Spaces and Places for Learning

Some valuable exceptions are beginning to offer new insight. Gannon and colleagues (2019: 49) describe how educators are ‘entangled with the concrete specificities of material spaces and objects’, and consider the interactions with others that arise in the ‘interstices of everyday academic life’. Similarly, in work on student engagement, Gourlay and Oliver (2018) explore how a broader understanding of experiences as embodied, socially situated, and taking place in complex networks — that we might conceptualise as *assemblages* — can enrich our knowledge of learning experiences. Gravett (2022) explores how attending to the material in higher education might enable us to look anew at learning and teaching as sociomaterial practices, whilst Gourlay (2022: 60) examines how we might employ concepts of social topologies and fire space to understand the ‘complex and multifaceted nature of space and presence in digital education’. Boys (2021, 2011) also attends to the spatial and material practices of teaching and learning. Specifically, her work considers the value of understanding how staff and students negotiate the ‘entanglements of their own lives with teaching and learning practices and how the pandemic has opened this up to critical view’ (2021: 22). Likewise, Mulcahy (2012) examines how teacher professional learning can be conceptualised as ‘constituted and enacted by people and tools in complex collectives or assemblages’ (2012: 133). For Mulcahy, ‘this learning assumes different forms and creates different knowledge and identity effects in different locations’. Similarly, McCune (2018: 318) argues for attention to be paid to the situated social and material practices of academic colleagues, rather than simply seeing teaching in terms of decontextualised knowledge and skills. Interesting work by Jandrić and colleagues (2020, 2021) explores photos and testimonials from specific moments during the pandemic, surfacing the challenges experienced by teachers and students.

Nonetheless, a relative absence of literature attending to the spaces of educators exists in comparison to the student learning space literature, although valuable insight can be gained from this body of work. For example, Lamb and colleagues (2022) continue conversations regarding the evolving relationship between digital technologies and university learning spaces. They explore the value of engaging a sociomaterial sensibility, which they contend ‘discourages us from conceptually reducing a learning space to its physical dimensions and contents … Instead, we are able to recognise a learning space as contingent on a complex and shifting assemblage of human and non-human actors.’ (2022: 3) In work by Bayne and colleagues (2013: 581) on the social topologies of distance students, the authors engage Fenwick et al. (2011: 153) to explore how institutions can be characterised by ‘flux and flows’, and to suggest that more research is needed into how ‘re-worked institutional social topologies are experienced’.

Like other authors cited above, we also work with sociomaterial theoretical approaches that enable us to understand education as assemblages, materialities, and processes, and draw our attention to the significance of ‘material stuff and spaces’ (Fenwick et al. 2011: 7). And we draw upon posthuman concepts — for example in Barad’s work (e.g. 2007). Using the notion of *entanglement*, Barad demonstrates how rather than existing as separate entities, individuals can be understood as being entangled with their surroundings (both human and non-human): ‘intra-actively constituted through the material-discursive practices that they engage in’ (2007: 168). In this article, we also engage Massey’s (2005) approach to understanding space and place as relational. For Massey, spaces and places are ‘practices of material engagement’ (2005: 61). Whilst places can be conceptualised as a situated ‘throwntogetherness’ (2005: 140), as ‘integrations of space and time’ and as ‘spatio-temporal events’ (2005: 130), spaces are ‘a simultaneity of stories-so-far’ (2005: 12), a rich and fluid constellation of interactions, a simultaneity of many stories. These conceptions offer an irruption to notions of space and place as flat, neutral, and fixed. Instead, space is depicted as a multiplicity of experiences. Space and place are not distinct, but rather entangle together, and offer mobile and valuable concepts for our understanding of relational connections.

## Engaging Photovoices

In order to think in new ways about the spaces and places for conversations and connections in higher education, we adopted an approach inspired by the rich tradition of photovoice research. As explained in the introduction to this article, our study was undertaken during the Covid-19 pandemic — and at a time when the pandemic was bearing some of its strongest effects. In our own working contexts (in the UK), mirroring the situation in many other countries, almost all campuses were closed, with teaching migrated entirely online. University staff made rapid changes to learning, teaching, assessment, and student support, whilst students studied remotely. Whilst these drastic changes led to some notable innovations in pedagogic practice, they also bore unequal consequences for students and staff, depending on their domestic circumstances and access to technology. For most, it removed the opportunities to teach, learn, or meet in ‘conventional’ places. This wider backdrop, and the need to work and learn in new spaces and places, represents influencing factors in both the focus of our study and our decision to adopt our photovoice method. The next section expands on how we conducted our research, and our use and implementation of a photovoice-inspired approach.

Photovoice has been defined as a visual approach which holds value for eliciting different and additional information from that which can be gleaned by more commonly adopted research methods such as interviews (Wass et al. 2019). Originating from work by researchers Wang and Burris (1997) to describe the method they adopted for illuminating the experiences of rural women in China, the primary aim is to include participants actively within the research via the inclusion of their own photographs (and ‘voices’). As such, it is a method which ‘asks participants to take photographs of things they associate with and/or practice as part of the community to which they belong, and thus give “voice” to their collective experiences’ (Waight 2020: 180). Photovoice differs from photo elicitation in its engagement between participants and researchers throughout the process, and its invitation to participants to actively share their own photographs, as opposed to the definition of photo-elicitation as ‘the insertion of a photograph by the researcher’ (Shaw 2021: 337). In terms of our own research context, we considered the scope of our study to be the diverse, international, community of teachers working in higher education (a community to which each of the authors also belong). We were keen to create a space for a diversity of teachers to share their photographs, experiences, and voices. As researchers, we wanted to expand our gaze beyond the social and the dialogic, and therefore photovoice also offered an approach to enable us to better understand the sociomaterial assemblages (Gourlay and Oliver 2018) that surround and form the spaces and places of higher education. We were interested to know what such interactions might look like; how might they be materially felt? Including photographs as a key part of our dataset would, we believed, enable us to reconsider the material contexts of learning and connections.

Ethical approval was gained from the principal investigator’s institutional ethics committee. Following this, 13 academic staff (see Table 1) accepted our invitation sent via social media (Twitter) to participate. As the focus of our study was upon the spaces for dialogue as identified by participants, as opposed to a granular analysis of how these choices may vary depending on the teacher’s experience, gender, etc., demographic and further participant information was not requested from these participants. However, based on the self-reported information, we know that our participants formed a diverse group that included teachers predominantly from the UK, but also from countries including Turkey and Japan. We also know that some participants were experienced academics, whilst others were new to academia and were teaching part-time alongside their doctoral studies.

**Table 1:** Pen portrait of the participants

	**Professional profile as indicated by participants**	**Focus of the conversations**
Participant 1	Senior Lecturer (British academic engaged in educational development)	Teacher and student interactions
Participant 2	Senior Lecturer (working in a British University)	Researcher and teacher interaction
Participant 3	Associate Professor in University Pedagogy (Norwegian academic teaching both students and staff)	Teacher and student interactions
Participant 4	Professor of Economics	Student interactions
Participant 5	Research Assistant (working with student teachers on their graduate research projects in Turkey)	Teacher and student interactions
Participant 6	Three generations of a family — the participant herself (Professor) who captures the conversations with her daughter, a British Sign Language (BSL) teacher and sign theatre performer and interpreter, and her other daughter, a doctoral student and BSL university lecturer.	Teacher interactions
Participant 7	Lecturer (working in a British University)	Teacher interactions
Participant 8	PhD Student (located in Japan and recently completed PhD in a Japanese university)	PhD students on reflection of his/her spaces for connection — a classroom/seminar room
Participant 9	Academic Developer and EdD Student	Teacher interactions
Participant 10	Educational Developer and Assistant Professor	Teacher and student interactions
Participant 11	Not specified	Teacher interactions/research connections
Participant 12	Not specified	Student interactions
Participant 13	Lecturer in Biology	Teacher-student and student-student interactions

Following an expression of interest to participate in the study, prospective participants were asked to provide consent. Participants were then provided with written information which invited them to take one or more photographs of everyday spaces that captured a learning and teaching conversation or encounter that had been meaningful to them. Specifically, participants were encouraged to consider the role of spaces and places in creating opportunities for connection and professional development. Participants were invited to contact the lead investigator with any questions and were given 1 month to respond. Whilst the majority submitted just one photograph, five chose to submit multiple images.

Additionally, participants were asked to write a short reflective narrative of approximately 500 words to accompany their photograph/s, drawing out the meaning of their image(s) and articulating the value to them in terms of its capturing of a space in which a meaningful learning and teaching encounter had taken place. The aim of the requested narratives was to surface participants’ own voices upon the image/s they had shared: particularly how the image/s represent a space for connection and how this is valuable to them in their development or practice. The images therefore became the reference point for participants to elaborate on the spaces they identify as promoting connections. In view of informed consent and anonymity procedures, participants were instructed to not include other individuals in their photographs. They were also advised that they could withdraw from the study at any time and that their participation was voluntary. Following the writing of this article, a further stage was carried out where participants were invited to review the paper, and specifically to comment on how their photographs and reflections had been presented. They were encouraged to review our interpretations of their contributions and the findings identified, and to elaborate or edit as necessary. Following this stage, minor proofing changes were made in view of participants’ comments.

## Data Analysis

Our data corpus — the participant narratives and photographs — was analysed inductively using a thematic approach (Braun and Clarke 2006). Firstly, all four researchers read through the data set and familiarised themselves with the reflections and photographs, noting initial ideas, interpretations, and potential themes. Secondly, the researchers met on three further occasions to share thoughts, to refine and to reach agreement regarding themes via discussion. Our visual data — the photographs — informed the themes and were analysed alongside the reflective pieces. At this stage we found Rose’s work on visual methodologies and materials helpful (2007, 2014). Rose (2014: 28) explains that participant’s generated visual materials are valuable in exploring elements of research participants’ lives that might otherwise be taken-for-granted; asking participants to take photographs of their lives, and then to talk or write about the photos, involves reflecting on activities in ways that are not usually done. We were interested to look again together at the taken-for-granted aspects of educators’ lives. Rose also explains that visual research methods are ‘heavily invested in the everyday … which creates a social of the ordinary rather than the extreme … photographs are understood as traces that elicit affects beyond talk’ (2014: 30). As a result, we wanted to consider what are the affects and ideas that the photographs elicit? How does our data surface the everyday? Following our analysis, we identified four interwoven themes across the dataset. Finally, the authors’ analysis of the data and our subsequent findings were shared with the participants who were invited to edit and expand as appropriate.

## Reflections and Limitations of the Study

Adopting the photovoice method enabled us to collect a rich corpus of reflections and images. However, during our own evaluation of the study, we also considered how creating additional spaces for dialogue with participants, for example via interviews, co-authoring, or further collaboration opportunities, would enrich future work. Our participants brought a diversity of experiences, locations, and roles to the study; this added value to the research that we had not foreseen. However, on reflection, requesting additional demographic detail, as well as information regarding other people included within reflections, could have enhanced the meaningfulness of our data.

## Findings

Participants’ chosen photographs represented a breadth of different spaces, places, and interactions including outdoor meetings, meeting rooms, offices, a laboratory, classrooms, kitchens, a cafe, and a university lobby. Our four main findings are now discussed below, with participants identified as Participant 1 (P1), Participant 2 (P2), and so on.

### Affective Connections

**Fig. 1:**
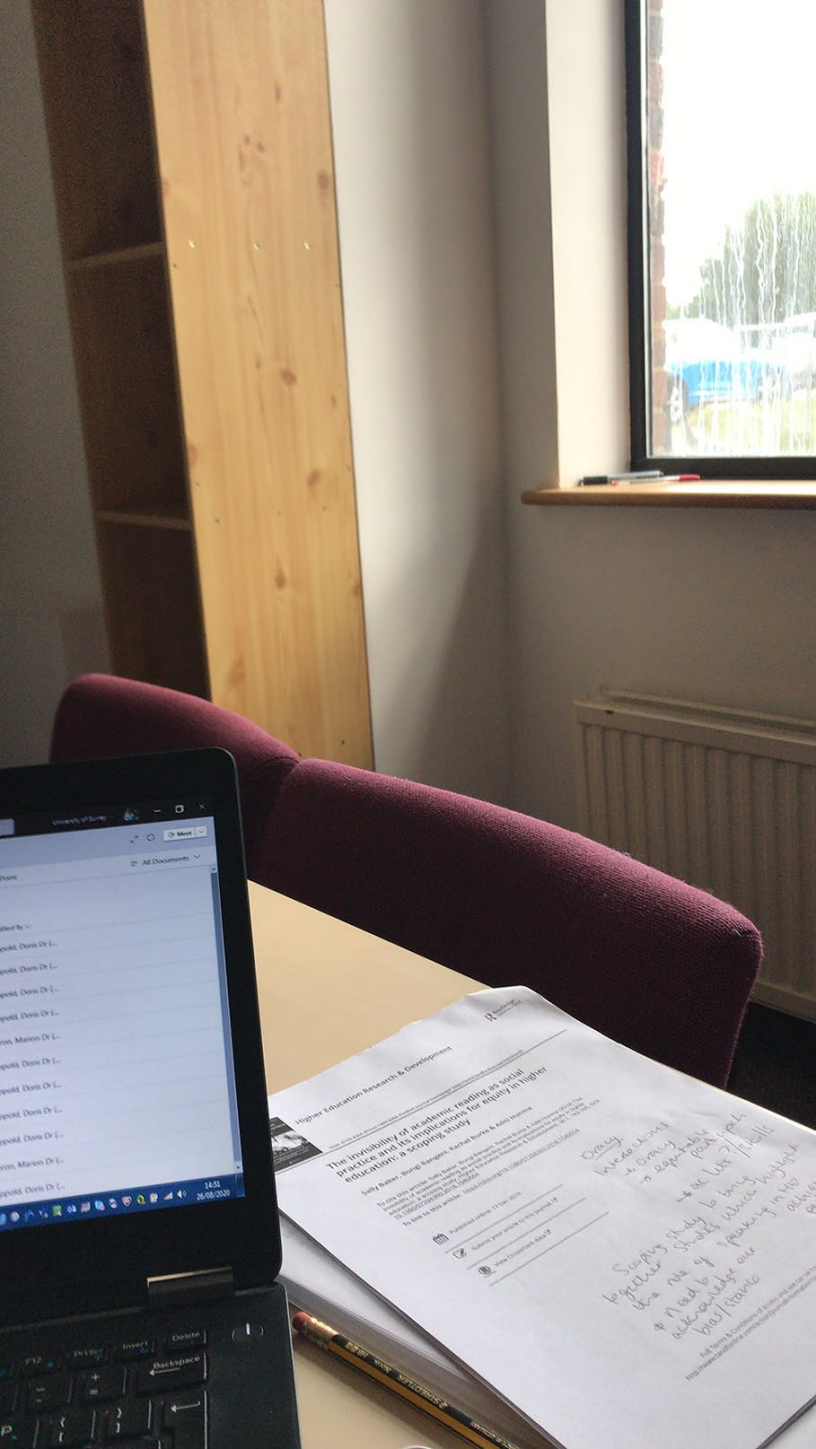
A ‘very bland and plain [meeting] room’ as described by P2

Participants’ data provided insight into the affective and emotional nature of academic life. Participants recurrently reflected on the joy and pleasure of connections, as well as the opportunities for developing trust, creating hope, and the power and value of such interactions. One example of this is P2, who describes collegial connections occurring during meetings about educational research, within a ‘very bland and plain [meeting] room’ (Fig. 1). This participant comments on how what is significant about the space is the joy, laughing, and energy created by the people in the room:

[T]his room is an example of how a seemingly uninspiring room can generate great ideas due to the dynamism and energy from the people in that room. As a team, we have laughed a lot in that room, had serious discussions and generated ideas. Rooms are important, places are important, but ultimately, it is the people in those places who create the energy.

The power of connections is also explored by P6 who describes the ‘energy that I drew from accidental conversations about teaching and learning’. Whilst P1 considers the significant value of learning and teaching conversations:

In my role I have almost daily conversations with teachers about the complex interrelationship between learning and teaching ... I have become increasingly aware that there is significant value in the shared experience and the dialogue that occurs, enhanced by stronger connections that enable great vulnerability to be surfaced.

**Fig. 2:**
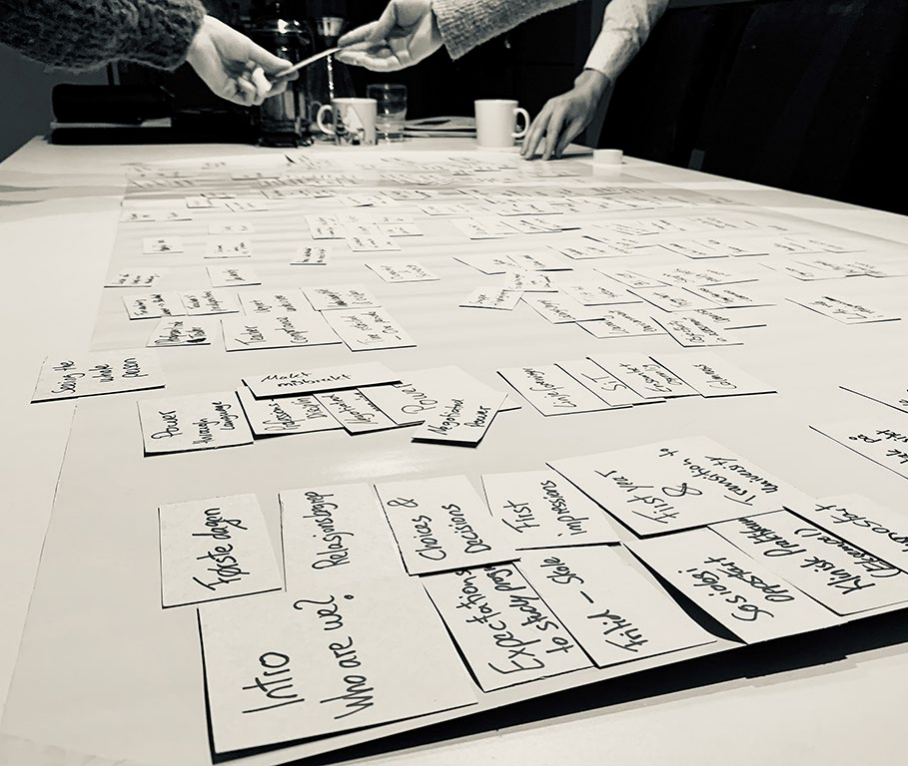
Discussion of a data analysis session with students

In their discussion of a data analysis session with students (Fig. 2), P3 articulates how such connections with others can generate a sense of hope: ‘we explore our different perspectives on today’s higher education landscape and re-imagine higher education as a place of hope’.

**Fig. 3:**
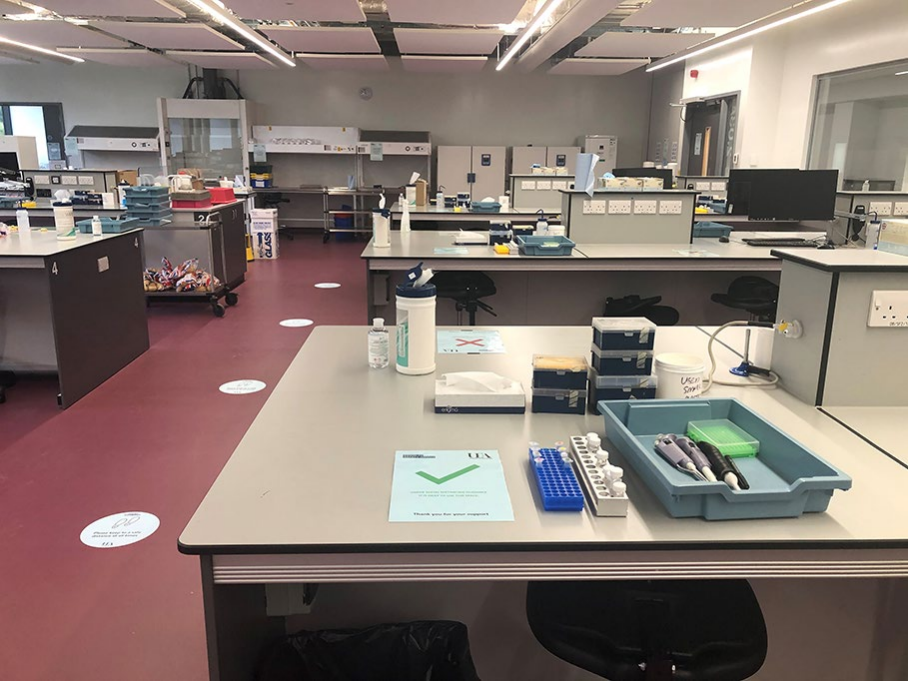
An image of a laboratory where staff and students meet and learn together

For P13, connections and conversations present an opportunity to reduce hierarchical structures and foster trust. P13 includes an image of a laboratory (Fig. 3) where staff and students meet and learn together:

[B]arriers are broken down in the laboratory. We are all scientists wearing the lab coat uniform, interactions may be fleeting so they are more casual. The dialogue is low stakes, the lab is noisy (so not everyone can hear your questions). Conversations are casual, it’s OK to giggle, it’s OK to ask for help, it’s OK to learn! There is time and space for reflection, time to get to know each other and to build trust.

**Fig. 4:**
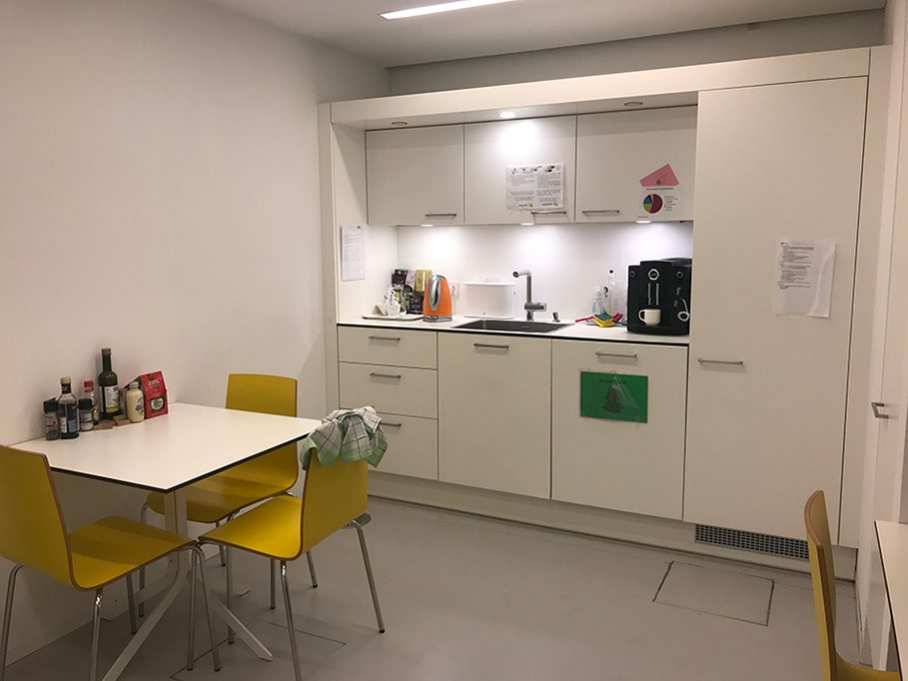
Tea kitchen, a place where you meet and catch up with colleagues from other institutes, scholars from different hierarchical levels, and a space for friendships to develop

Likewise, P11 also describes how spaces and connections can lead to an important breakdown of hierarchies and the building of trust:

The tea kitchen [Fig. 4] is a place where you meet and catch up with colleagues from other institutes, scholars from different hierarchical levels and a space for friendships to develop: a space for informal talks about learning and teaching − we exchange concepts and exercises, plan lessons together, talk about problems like discipline in the seminar rooms ... naturally we also switch back and forth from private topics to teaching and vice versa.

Together, these excerpts and images surface the diverse spaces and places that enable and represent connections in learning and teaching, as well as how such moments of academic life support teachers’ development. The examples offer glimpses into the affective and discursive encounters between teachers and colleagues, and teachers and students, and surface the emotions that characterise academic life: hope, joy, energy, and power. These findings resonate with the work of Thomson and Barrie (2021) who highlight the power of informal conversations about teaching to foster camaraderie, to build friendship and provide support. Similarly, they build upon work by Cook-Sather and colleagues (2021) who surface the role of faculty-faculty and student-faculty conversations in developing voice, agency, and trust. Our findings also resonate with Gannon et al. (2019: 50) who attend to ‘the affective attunements of joy’ and ‘subtle shifts in atmosphere’ of academic life, contending that ‘in these moments different energies are released, different attunements made possible’. As P2 explains, ‘as a team, we have laughed a lot in that room’.

### Nature, the Outdoors, and Well-Being

**Fig. 5:**
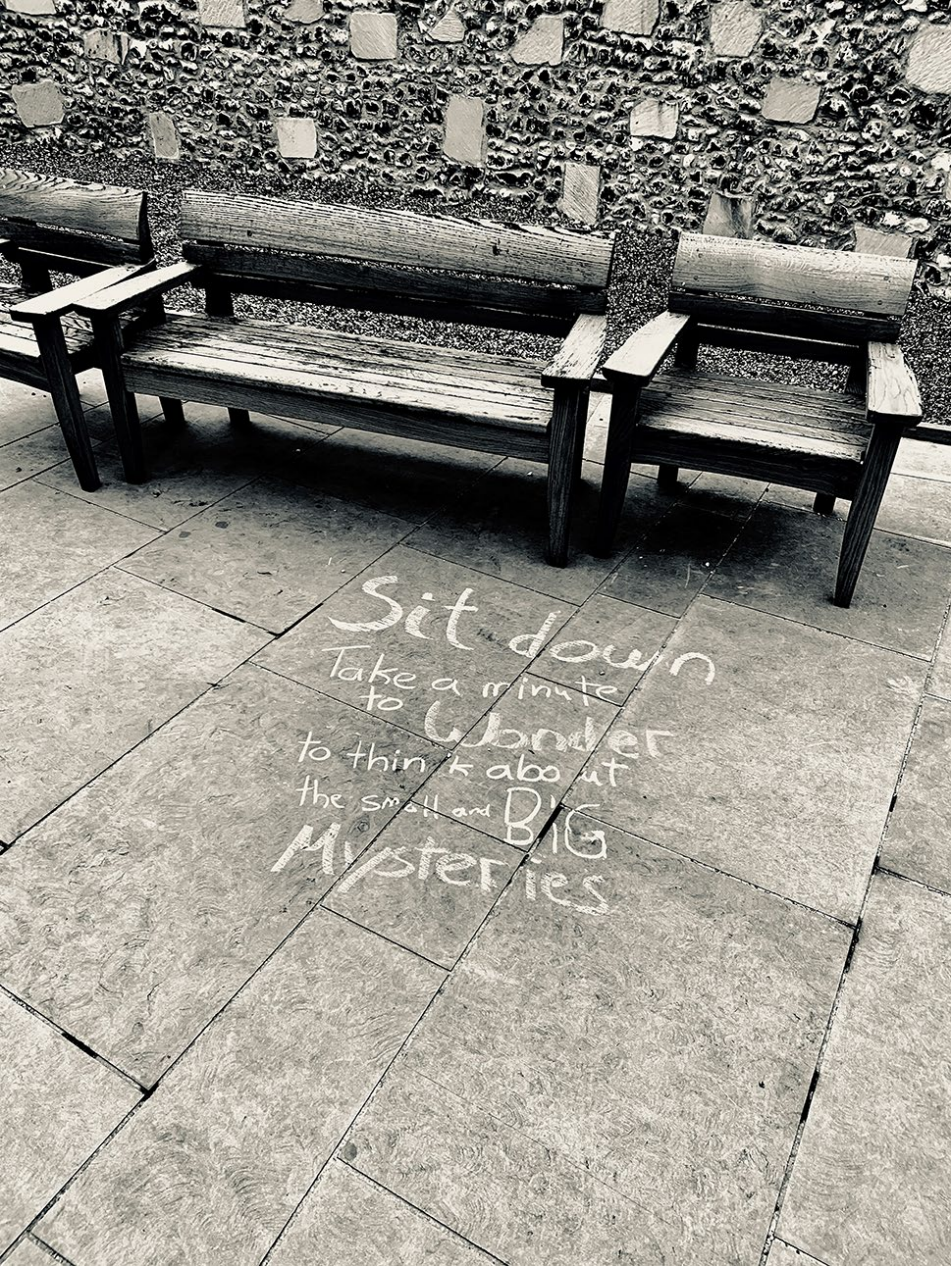
P3’s photograph taken in an English city during a conference

Participants also explored the relationship between the permeability of the university’s spaces and its boundaries within their photographs. In the following image, P3’s photograph (Fig. 5) is taken in an English city during a conference. This participant writes how he has started to explore possibilities to reclaim public spaces and take work outside of the physical boundaries of universities.

**Fig. 6:**
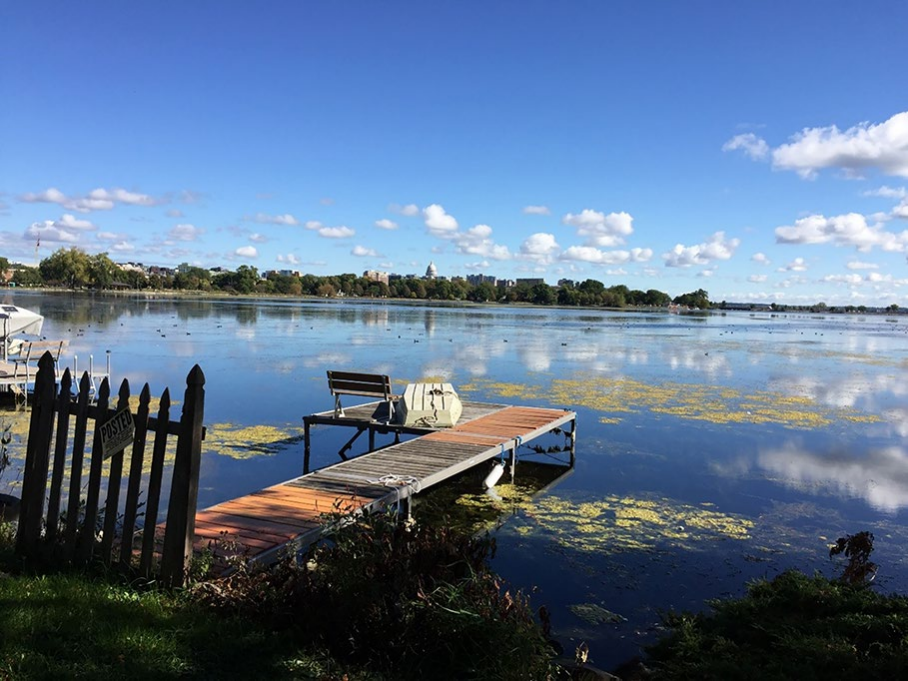
P1 chosen location as he explored how, in his view, the meaning of connections ‘becomes more apparent after they have finished’

Similarly, P1 chose the following location (Fig. 6) as he explored how, in his view, the meaning of connections ‘becomes more apparent after they have finished’. He writes of the fluidity of connections and how it is in spaces beyond the university that ‘I continue processing what this conversation provided ... free of the associations of an institutional setting that may challenge the values I want to focus on’.

**Fig. 7:**
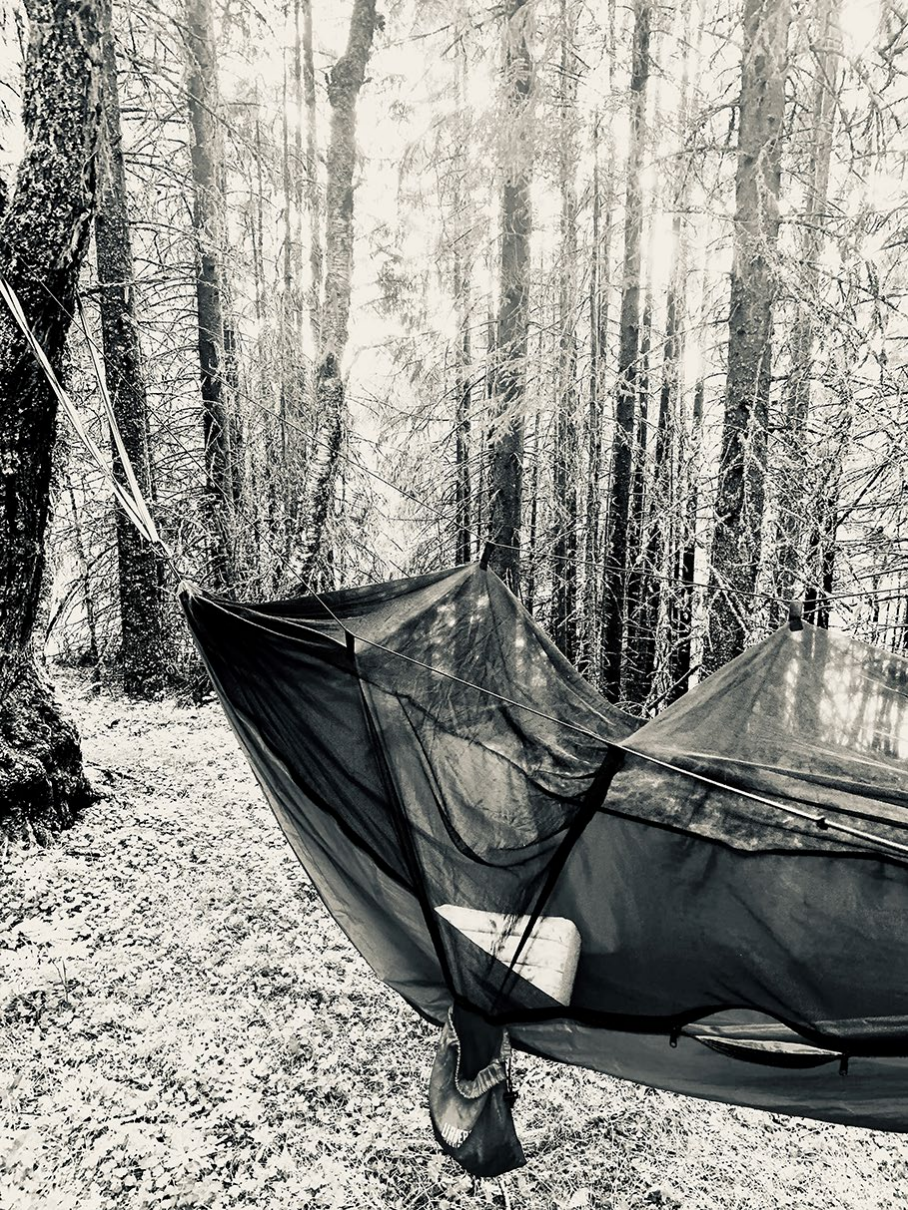
P3’s picture of a hammock in a forest, ‘where digital meetings take place with people all over the world to discuss ideas about higher education, teaching, and learning’

In another outdoor image (Fig. 7), P3 presents a picture of a hammock in a forest: ‘where digital meetings take place with people all over the world to discuss ideas about higher education, teaching, and learning’. P3 explains that this is

[A]n interesting space, where I on the one hand was alone in the forest, but at the same time I was connected to many other people. I believe that the grounding in nature provides an important element in this learning space and shapes the experience of learning in a profound way.

In recent work, Thomson and Barrie (2021: 331) discuss how the usefulness of conversation ‘may be vulnerable to the serendipitous nature of physical proximity. Some conversations did not happen, and some topics did not get raised because academics were not co-located in time and space.’ In our data however, participants recurringly reflect upon the fluidity of spaces for connection as well as the affordances of remote or alternative locations. Here, we see a diversity of spaces and places that enable and represent connections in learning and teaching. We can also begin to understand the complex and shifting nature of educational practices, as dislocated from the classroom or conventional spaces of learning. The varied locations chosen by participants suggest a myriad of spaces in which to connect, both within and beyond university campuses, despite the constraints of post-pandemic life.

### The Professional and Private: A Blurring of Boundaries

The blurring of boundaries between the professional and the private was also a salient theme that was visible across the narratives. P3 reflects upon a photograph of students working together in his own home in his kitchen (Fig. 2):

[W]hile being at home allowed us to cook together and work in a more different way than in a more formal office environment setting, it also poses a blurring of boundaries between professional and private spaces that potentially can create problems and be experienced as inappropriate and demanding.

**Fig. 8:**
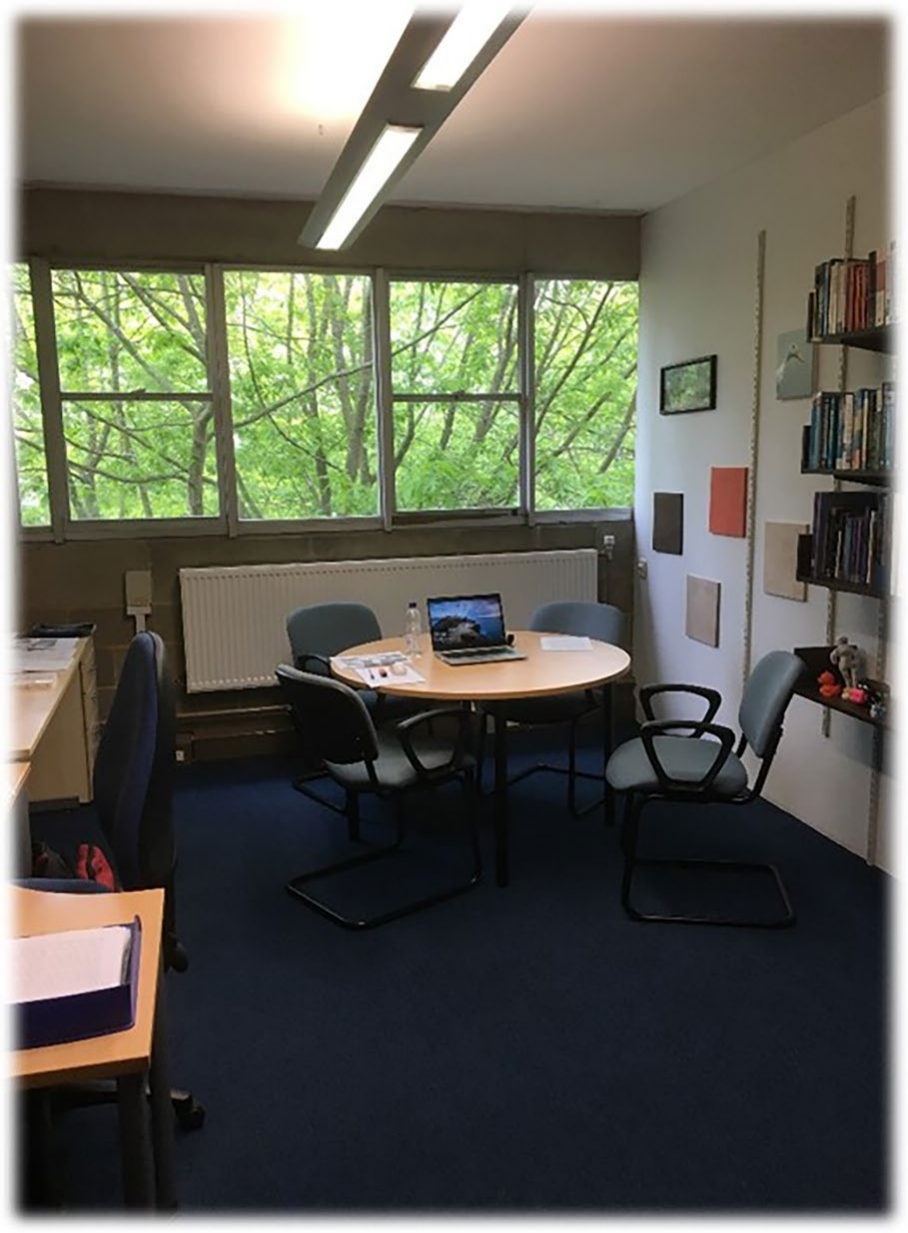
P4’s office where he meets students virtually

In contrast, P4 describes the pleasure of meeting students in his office (Fig. 8) and the enjoyable muddling of boundaries:

My office is a cosy environment: you can see pictures of ex-students at school socials, pictures of my family and friends, and various items talking about my story and my experiences working in different institutions. Students ask questions about all of these, and it is nice to have an exchange with them where we can recognise each other as humans, with a story, a life, dreams and aspirations.

For this participant, the mingling of pictures, stories, and other material items represent the blurring of the personal and the professional in a generative way: ‘where we can recognise each other as humans, with a story, a life’.

**Fig. 9:**
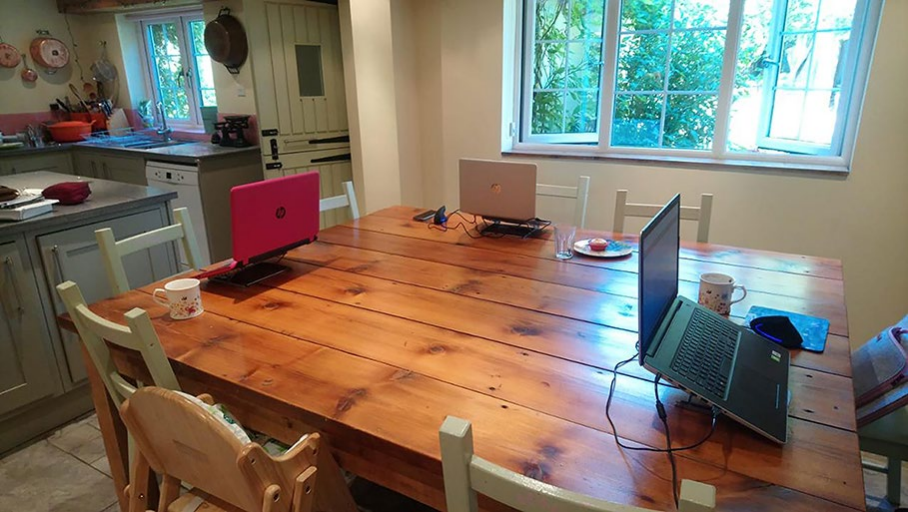
P6’s space created during lockdown

P6 explored how lockdown created a space (Fig. 9) for her to connect with her family as colleagues and have generative conversations about learning and teaching:

My family live in three, interconnected cottages ... I have been interested to see how space for teaching dialogue has developed in this place over recent months. My eldest twin daughter is a British Sign Language teacher ... Her younger twin is a British Sign Language lecturer at my university ... So, family dinners frequently include conversations around pedagogy, but the focus and sustained nature of these conversations has been noticeably enhanced by our lockdown situation. The three of us work around one kitchen table.

P6 continues to describe the interesting affordances of this ‘rather unexpected way of working’:

From our perspective, this rather unexpected way of working has several benefits. Technical glitches were fixed very quickly, because one of us usually knew the answer. If we needed the university's technical support team to help us with an online appointment, we all got the benefit if we wanted to listen in.

We crashed each other’s meetings from time to time. I now feel that I have a wider network for future teaching and learning conversations.

We talked about the technology we were using, or considering using. This exposure to different online teaching technology is something I would not have had without our kitchen table workspace. We also give CPD talks on online teaching and learning, so we have been able to listen in to each other when these are running.

For, P6 her kitchen table workspace has offered a positive alternative to the conventional spaces of university buildings:

I look forward to seeing my colleagues again, and the accidental conversations in the print room, but for me and my family, our teaching and learning lockdown has been a good one.

**Fig. 10:**
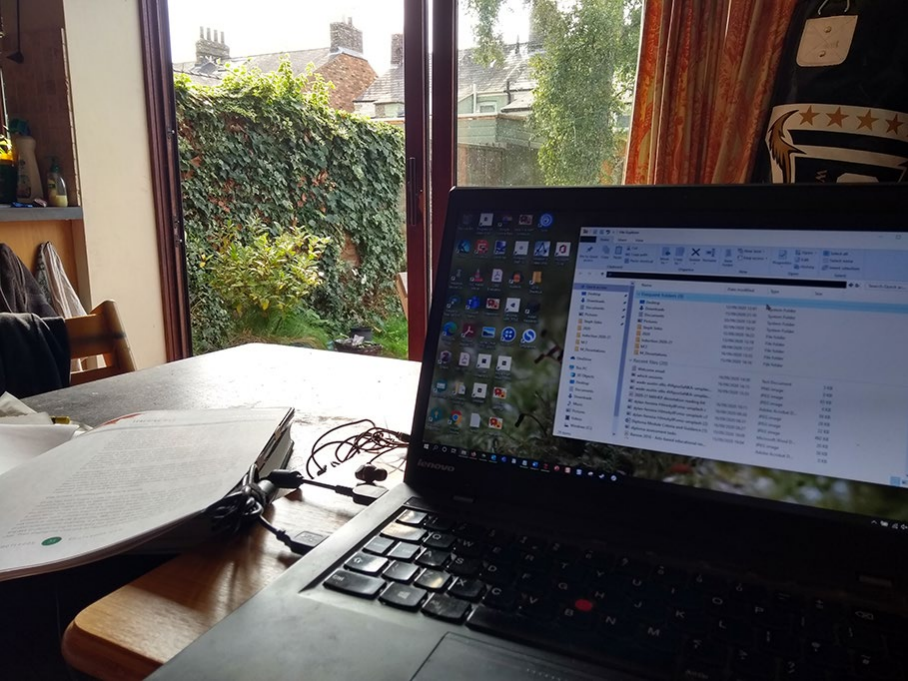
P9’s kitchen table

Similarly, P9 also included a picture of a kitchen table (Fig. 10).

I took this photo to show how the personal and the workplace are superimposed in my ‘new normal’ workday. The kitchen table is included in the photo as this is also where mealtimes with my family take place and where I paint or sew … I sometimes share this view of my workspace (or of my cats, small objects, or paintings) when talking to colleagues while video-conferencing as a way of personalising online interactions. I find this important as a kind of proxy for the small personalised comment that I used to make when meeting colleagues on campus (‘amazing pen’, ‘nice shirt’, ‘like the new earrings’). So I am learning to offer different kinds of tokens of social presence to help anchor interactions in who we are as ‘real’ people living in ‘real’ physical spaces, rather than digital postage-stamp faces. I miss conversations with colleagues in cafeterias or in their offices. The sociomateriality of the dialogues we have now is much more limited: no chance meeting on the walkway, no walking meetings. Placing boundaries between what is work and what is home can be tricky ... so small rituals such as sipping tea in the garden or going for a walk help me mark the end of the working day. I am an academic developer no matter where I work, online or in classrooms, but sometimes I want to feel at home, really at home.

Here, the description of his colleagues ‘digital-postage stamp faces’ is evocative in its depiction of a specific form of post-Covid interaction. As a result of the move to online learning and teaching educators are learning to offer different kinds of ‘tokens of social presence’, in order to help ‘anchor interactions in who we are as “real” people’. Further, this participant describes how they have erected new boundaries to separate off the home and work spaces, to prevent blurring, and to recreate a sense of home free from the intrusion of the work of academic life. These extracts and images expose both how professional learning occurs within porous and fluid spaces, as well as the complex and shifting nature of educational practices, as a result of the altered post-Covid higher education landscape.

### Material Matters

**Fig. 11:**
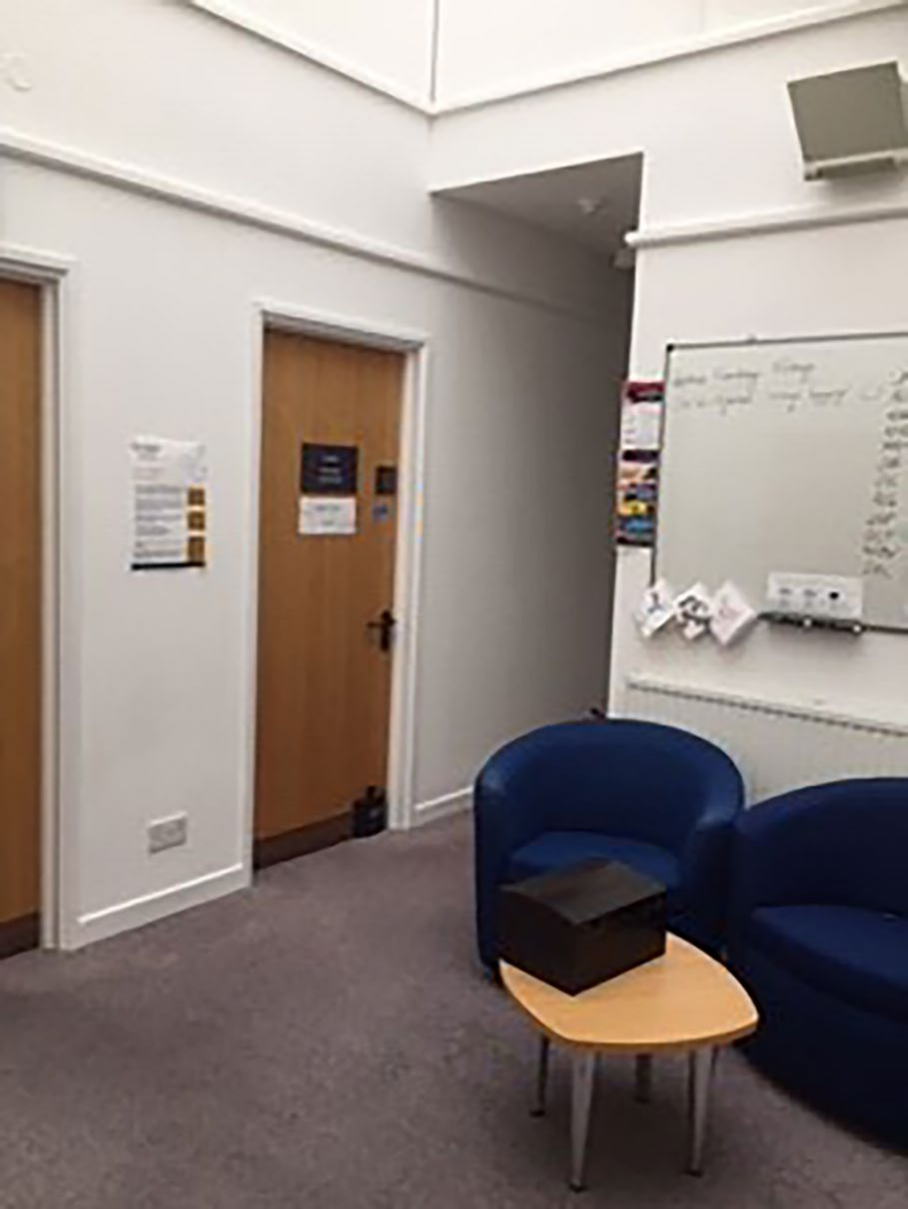
P7’s picture of university building lobby

Our data show vividly how spaces and the material intra-act (Barad 2007) with the affective, social, and discursive elements of academic life. We have seen how for P9 their experience is bound up in the presence of the kitchen table, their cats, small objects, and paintings. For them, this represents the new version of commenting on the physical in person: ‘amazing pen’, ‘nice shirt’. We have also seen how spaces represent and create connections. P7 describes this powerfully with his choice of a university building lobby (Fig. 11). The lobby

connects up all of the offices of the colleagues in my team. I have chosen this because some of our most useful and fun discussions take place in and across this space. Sometimes we are in our individual offices and colleagues ‘shout’ between rooms, so our conversations have to cross this space. Some of these discussions can simply be practical (e.g. who is teaching when, what room, etc.), but at times we congregate in this space to have lengthier and more meaningful discussions. For example, sometimes I have returned from teaching to find a colleague outside an office, so we might have a simple informal debrief about a teaching session and this can provide some useful unplanned feedback. Sometimes there can be an informal chat between a couple of colleagues in this space, so we overhear things and decide to come out of our offices to join in ... it is the informality of the space that is what makes this important to me – the fact that this isn’t an office or meeting room means that conversations are nearly always unplanned. We are all siloed off in our separate offices, so we need to literally enter this space to come together ... As a result of the pandemic forcing us to work at home, most of us have been away from this space for some time now. I feel this has emphasised how valuable this space was for my learning.

**Fig. 12:**
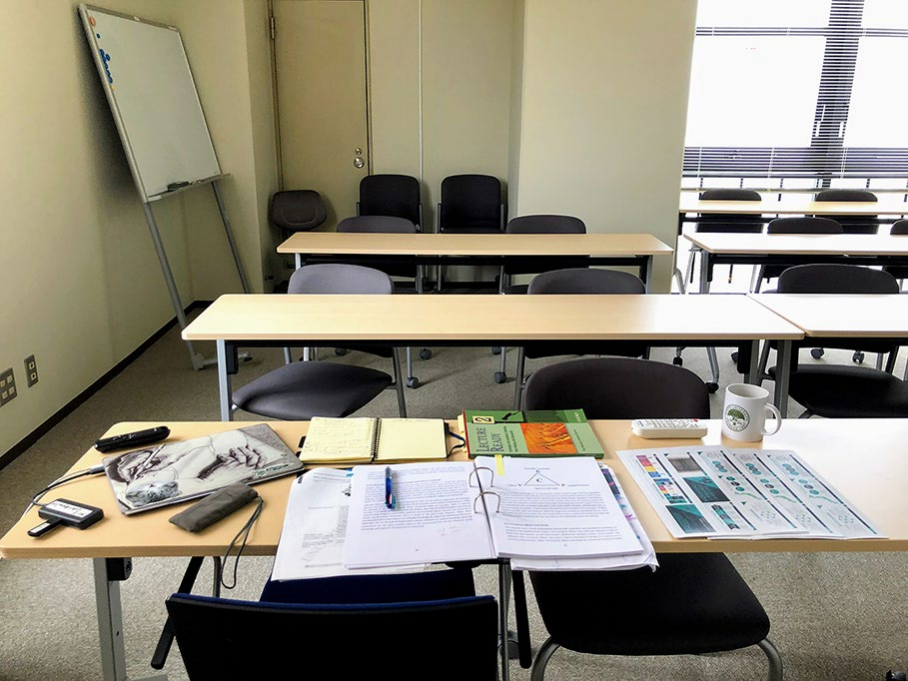
P8’s picture of a particular classroom showing how objects fill the table that each hold meaning to her

From a different perspective, P8 discusses connections solely between herself and the space. The significance of objects form part of her connection. Describing a particular classroom (Fig. 12) she writes how objects fill the table that each hold meaning to her.

My University mug, my trusty laptop, and my notebook of abandoned and half-baked ideas. The room itself holds meaning. The whiteboard in the back corner ... the window in the back overlooking the incredible mountains ... the light, musty scent that reveals the age of the building. The picture is dull to most that see it but to me it is alive.

## Discussion

Our data have exposed the myriad of physical spaces and places where connections can occur and how such spaces offer ‘affective attunements’ (Gannon et al. 2019: 50), enabling joy, laughter, trust, hope, empowerment, and friendship to be felt. Such experiences offer sustenance, re-energising, and supporting teachers to continue with their work, as well as to learn. Our study offers value in identifying the importance of learning and teaching encounters as an integral aspect supporting teachers’ feelings of connection and well-being, and highlights the value of attending to learning spaces for educators, as well as for students. However, notably, our data also surfaced the permeability and fluidity of spaces, how learning can exceed the boundaries of a specific space, spilling over into spaces beyond the original location (P1; P3). Engaging sociomaterial and spatial theories (Massey 2005; Fenwick et al. 2011) enables us to see how spaces can be understood as ‘flux and flows rather than simple bounded space’ (Fenwick et al. 2011: 153), as relational, and as a multiplicity of experiences. As P3 explains, ‘on the one hand was alone in the forest, but at the same time I was connected to many other people’.

Adopting a photovoice approach enabled us to explore the messy contexts and practices of education, enabling rich and nuanced stories to emerge. McCune (2018: 319) explores the value of such approaches: ‘educational development practice which engages deeply with messy realities may be more likely to access the points at which pedagogic practices can shift in valuable directions’. Our study joins a growing body of work that foregrounds the particularities of localised learning environments — as opposed to generalisable practices. Instead, ‘studying teacher learning and teacher knowledge in its material specificity’ (Mulcahy 2012: 134–135) is important if we are to understand the complex multiplicities of teaching practices — their ‘throwntogetherness’, and ‘simultaneity-of-stories’ (Massey 2005: 140, 12). Our study surfaces the materiality of such encounters, and specifically the intra-action of the social, affective, and material (Fenwick et al. 2011). These findings resonate with work which articulates learning spaces as shifting assemblages (Gravett 2022; Lamb et al 2022; Gourlay and Oliver 2018), or as ‘rich, tangled webs of the social and the material from which academics’ pedagogic practice in higher education emerges’ (McCune 2018: 319).

This fluidity of spheres and practices suggest a need to move away from binary conceptions of the digital and in person, and a need to smudge the lines of those enclosures of thought that linguistically common expressions — ‘remote’, ‘in person’, ‘face to face’ — surrounding the digital impose. The implications of this are that we can see educational practices as entangled (Barad 2007), as always constituted by digital activities, regardless of modality (Fawns 2019, 2022), and technologies as situated within sociomaterial assemblages. Changing practices warrant us to unbound our thinking; as Gourlay (2022: 59) describes, we are gathering:

growing insights into the sheer complexity, contingency, and shifting nature of educational practices and engagement in terms of spatiality, embodiment, and movement. These are crucial questions for scholars of higher education when considering both the campus, and also the nature of online engagement.

Our research also exposes the complex blurring of boundaries between the personal and the private, and the ways such a blurring may be experienced when teaching within the postdigital university. For teachers working remotely and teaching online, the resultant mingling of personal and private spheres may offer surprising affordances, for example the opportunity to work more closely with family members. However, it may also be unwelcomed, with the lack of separation between home and work experienced as intensely problematic, requiring new strategies to erect some division between these spheres. Of course, such intrusions are also likely to be experienced disproportionately by different social groups, for example academics with caring responsibilities, and therefore can only add to social inequities and the difficulties academics face in managing the divisions between work and home. These findings resonate with Boys who articulates the value of understanding how staff and students negotiate ‘the complex entanglements of their own lives with teaching and learning practices’ (2021: 22).

## Conclusions

In this study, we sought to explore the spaces and places that enable connections in learning and teaching, to examine how such moments support teachers’ development, and to understand the complex multiplicity of educational practices — particularly in view of a higher education sector that is experiencing rapid change. Our article offers a number of contributions to higher education research. Engaging sociomaterial and spatial concepts and building upon literature that foregrounds the materiality of educational practices, our study provides insights into the materiality and fluidity of spaces and places in higher education. Crucially, it foregrounds the spaces in which teachers, rather than students, learn, surfacing the experiences of educators, what matters to their well-being, and how they may find interstices in which to experience connection. Our study also provides a means to examine teachers’ diverse experiences of the difficulties they have faced and to share how they have worked within and through challenges presented by the pandemic. As what it means to work and learn in higher education is rapidly evolving, this foregrounding of teachers’ experiences offers new insight into the pleasures and complexities of academic life, and exposes the value of attending to the specificities and messy realities of teaching and learning in higher education.
